# Achondroplasia manifesting as enchondromatosis and ossification of the spinal ligaments: a case report

**DOI:** 10.1186/1752-1947-2-263

**Published:** 2008-08-11

**Authors:** Ali Al Kaissi, Rudolf Ganger, Klaus Klaushofer, Monika Rumpler, Franz Grill

**Affiliations:** 1 Ludwig Boltzmann Institute of Osteology, Hanusch Hospital of WGKK and AUVA Trauma Centre Meidling, 4th Medical Department, Hanusch Hospital, Vienna, Austria; 2 Orthopaedic Hospital of Speising, Paediatric Department, Vienna, Austria

## Abstract

**Introduction:**

A girl presented with achondroplasia manifested as mild knee pain associated with stiffness of her back. A skeletal survey showed enchondroma-like metaphyseal dysplasia and ossification of the spinal ligaments. Magnetic resonance imaging of the spine further clarified the pathological composites.

**Case presentation:**

A 7-year-old girl presented with the classical phenotypic features of achondroplasia. Radiographic documentation showed the co-existence of metaphyseal enchondromatosis and development of spinal bony ankylosis. Magnetic resonance imaging showed extensive ossification of the anterior and posterior spinal ligaments. Additional features revealed by magnetic resonance imaging included calcification of the peripheral vertebral bodies associated with anterior end-plate irregularities.

**Conclusion:**

Enchondromas are metabolically active and may continue to grow and evolve throughout the patient's lifetime; thus, progressive calcification over a period of years is not unusual. Ossification of the spinal ligaments has a specific site of predilection and often occurs in combination with senile ankylosing vertebral hyperostosis. Nevertheless, ossification of the spinal ligaments has been encountered in children with syndromic malformation complex. It is a multifactorial disease in which complex genetic and environmental factors interact, potentially leading to chronic pressure on the spinal cord and nerve roots with subsequent development of myeloradiculopathy. Our patient presented with a combination of achondroplasia, enchondroma-like metaphyseal dysplasia and calcification of the spinal ligaments. We suggest that the development of heterotopic bone formation along the spinal ligaments had occurred through an abnormal ossified enchondral mechanism. We postulate that ossification of the spinal ligaments and metaphyseal enchondromatous changes are related to each other and represent impaired terminal differentiation of chondrocytes in this particular case. Standard radiographic examination showed spinal bony ankylosis only. The pathological composites of the vertebrae have been clarified using scanning technology. Extensive spinal ligament ossification associated with calcification of the peripheral vertebral bodies and anterior end-plate irregularities were notable. We report what may be a novel spinal and extraspinal malformation complex in a girl with achondroplasia.

## Introduction

Achondroplasia is the most common form of skeletal dysplasia characterised by short limb dwarfism. It occurs as a result of mutations in one copy of the fibroblast growth factor receptor 3 gene (*FGFR3*). More than 97% of patients have the same point mutation in *FGFR3 *and more than 80% of these are new mutations. The mutation, which causes an increase in *FGFR3 *function, affects many tissues, most strikingly the cartilaginous growth plate in the growing skeleton, leading to a variety of manifestations and complications [1-3].

Enchondromas are common, usually benign, intra-osseous cartilaginous tumours that develop in close proximity to growth plate cartilage. Pathological fractures can occur and when a joint is involved this may result in shortening of a limb. The primary significant factors of enchondromas are related to their complications [4-6]. Progressive vertebral fusion is a not uncommon radiographic entity in children, often referred to as the Copenhagen syndrome [7]. We describe a previously unreported combination of achondroplasia, metaphyseal enchondromatosis and ossification of the spinal ligaments.

## Case presentation

A 7-year-old girl was brought to the orthopaedic department because of mild knee and back pain associated with restricted spine mobility. She was born full term following an uneventful gestation. At birth her length was around the 3rd percentile, whereas her occipito-frontal circumference (OFC) and weight were around the 25th percentile. She was clinically and radiographically diagnosed as having achondroplasia. This was confirmed through the detection of the common mutation of *FGFR3 *for achondroplasia. The parents were of normal height, healthy and non-consanguineous. Clinical examination at the age of 7 years showed marked growth deficiency, -4 standard deviations, and her OFC and weight were around the 50th percentile. Craniofacially the head appeared large with frontal bossing, but with midfacial hypoplasia. The hands were short and broad with fingers exhibiting a three-pronged (trident) appearance. Movements of the thoracolumbar spine were limited, but movements of the cervical region were spared. No associated abnormalities were detected on examination of the nervous system, eyes, heart or abdomen. The results of full blood analysis, erythrocyte sedimentation rate and C-reactive protein were normal. Moreover, there were no laboratory data suggestive of endocrinopathies, hypophosphatasia and/or hypercalcaemia.

**Figure 1:**
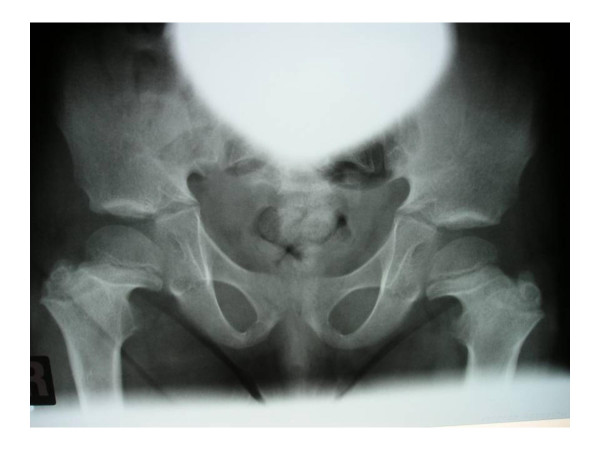
**Anteroposterior radiograph of the pelvis showing rounded iliac bones, a horizontal acetabular roof and small sacroiliac notes**. Coxa vara with defective modelling of the femoral necks associated with metaphyseal dysplasia with no trace of enchondromatous lesions.

**Figure 2:**
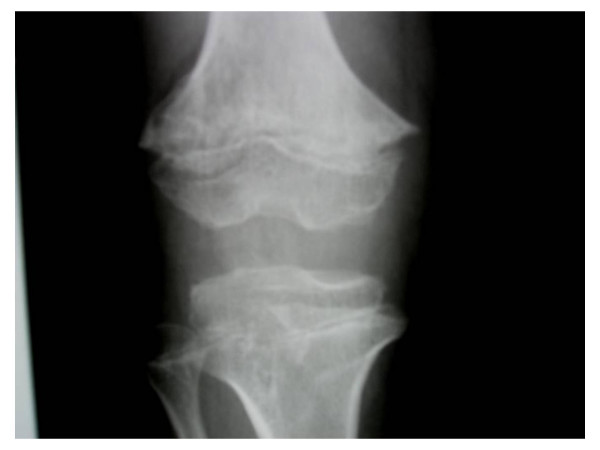
**Anteroposterior radiograph of the knee showing multiple small enchondroma-like metaphyseal dysplasias**. The distal femoral and the proximal tibial bones show metaphyseal cupping with multiple enchondromatous lesions and an abnormal metaphyseal trabecular pattern associated with small round rings and arcs and dense foci is as intended here within the metaphysis.

**Figure 3:**
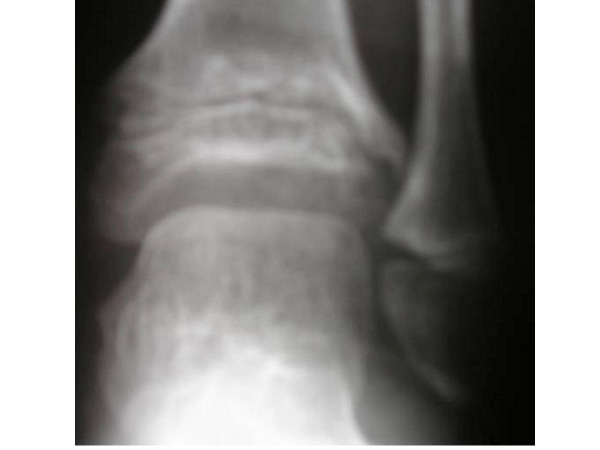
**Anteroposterior radiograph of the ankle joint showing enchondromas with the appearance of linear lucencies**. The chondrocytes appear to line up in a vertical orientation along the epimetaphyseal components associated with sclerosis of the articular surface.

**Figure 4:**
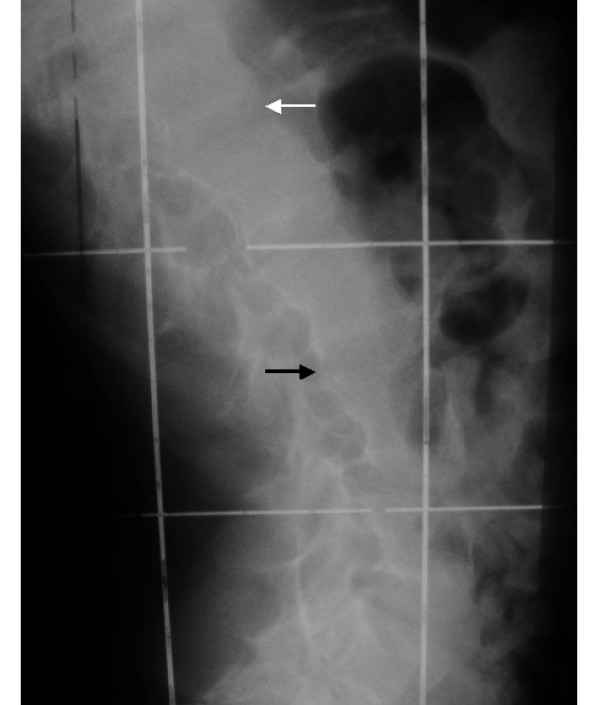
Lateral thoracic spine radiogram showing extensive ossification of the anterior (white arrow) and the posterior longitudinal ligaments with the development of long bony ankylosis with no skip areas along the posterior aspect (black arrow).

**Figure 5:**
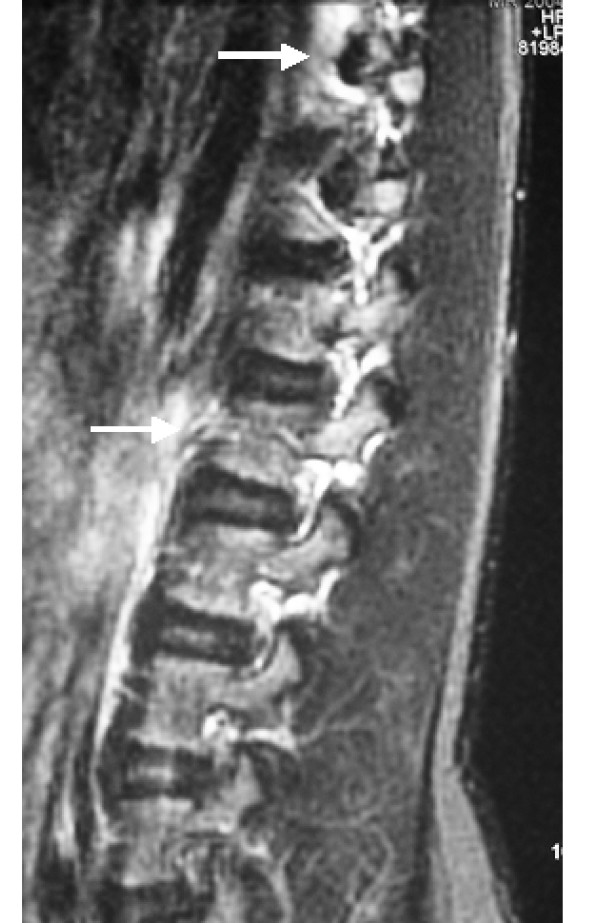
**Magnetic resonance imaging of the lower thoracic spine with sagittal T2 fast spin echo sequences showing the ossified anterior longitudinal ligament with subsequent anterior vertebral hyperostosis and bridging (arrows)**. In addition there was involvement of the posterior longitudinal ligament. Peripheral sclerotic borders associated with anterior end plate irregularities have outlined the overall vertebral bodies.

A skeletal survey and magnetic resonance imaging (MRI) were undertaken at the age of 7 years (Figures 1, 2, 3, 4, and 5). Achondroplasia is characterised by a long, narrow trunk and short limbs, especially in a proximal segment. It is the most common form of non-lethal skeletal dysplasia and the most common type of short-limb dwarfism [1-3] and is usually diagnosed at birth. Clinically the rhizomelic limb shortening and the broad and prominent forehead may not be striking, but radiologically the pelvis is clearly abnormal. The complications of achondroplasia involve many organ systems, but in most instances they are consequences of abnormal linear bone growth. About 10% of patients have tibial bowing by the age of 5 years which progresses through childhood, affecting 42% of adult patients. In adulthood and postadulthood periods the spinal canal size decreases with age relative to the size of the spinal cord, leading to lumbar spinal canal stenosis. One-third of patients with achondroplasia develop spinal stenosis requiring surgical intervention. However, this condition rarely develops before the age of 15 years [1-3,8]. MRI has been performed on a number of children with achondroplasia in order to study the aetiology behind the development of spinal canal stenosis [9]. Spinal ligaments were not included.

Enchondromatosis is a common bony dysplasia with a variable pattern of bony involvement. The well-differentiated forms of enchondromatosis are Ollier disease, Maffucci syndrome, metachondromatosis, spondyloenchondromatosis, dysspondyloenchondromatosis and genochondromatosis I and II [2,3].

Unlike other types of enchondromatosis, metaphyseal enchondromatosis is characterised by extensive development of enchondromas within the epiphysis before closure of the growth plate [2,3,10,11]. Numakura et al. [11] reported the cases of three boys presenting with achondroplasia and metaphyseal enchondromatosis. Nizankowska-Blaz and Kozlowski [10] reported the case of a girl with achondroplasia with knee pain secondary to metaphyseal enchondromatosis. Scanning techniques were not used in these cases, and spinal involvement was not seen. Frydman et al. [5] described the development of quadriparesis in connection with spondyloenchondrodysplasia. Spinal scanning was not used. Al Kaissi et al. [6] described progressive vertebral fusion in connection with spinal enchondromatosis in a girl without achondroplasia. The patient's father had been a patient at the rheumatology department because of thoracic spine bony ankylosis, even though all of his rheumatological tests had proven negative. They suggested that this father and daughter pair have a possibly distinctive form of spinal enchondromatosis associated with progressive ossification of the spinal ligaments.

## Conclusion

In summary, given the unusual range of malformation complexes in our present patient, it appears that standard radiographic documentation may be insufficient to further understand the composites of the spinal pathological mechanism. A referral to scanning technology is therefore recommended.

## Abbreviations

FGFR3: Fibroblast growth factor receptor 3; MRI: Magnetic resonance imaging; OFC: Occipito-frontal circumference.

## Competing interests

The authors declare that they have no competing interests.

## Authors' contributions

AAK was responsible for a) writing the MS, b) data analysis, and c) conception and design, KK and MR Participated in conception and design, FG Participated in data analysis.

## Consent

Written informed consent was obtained from the patient's next-of-kin for publication of this case report and accompanying images. A copy of the written consent is available for review by the Editor-in-Chief of this journal.
